# Repeated steroid injection and polyglycolic acid shielding for prevention of refractory esophageal stricture

**DOI:** 10.1007/s00464-023-10111-z

**Published:** 2023-05-16

**Authors:** Yoshiki Sakaguchi, Yosuke Tsuji, Junichi Sato, Dai Kubota, Miho Obata, Rina Cho, Sayaka Nagao, Yuko Miura, Daisuke Ohki, Hiroya Mizutani, Seiichi Yakabi, Naomi Kakushima, Keiko Niimi, Mitsuhiro Fujishiro

**Affiliations:** Department of Gastroenterology, Graduate School of Medicine, The University of Tokyo, 7-3-1, Hongo, Bunkyo-Ku, Tokyo, 113-8655 Japan

**Keywords:** Endoscopic resection, Triamcinolone injection, Polyglycolic acid sheet, Fibrin glue

## Abstract

**Background:**

Postoperative stricture and refractory stricture are severe adverse events which occur after expansive esophageal endoscopic submucosal dissection (ESD). The aim of this study was to assess the efficacy of steroid injection, polyglycolic acid (PGA) shielding, and of additional steroid injection thereafter for the prevention of refractory esophageal stricture.

**Methods:**

This is a retrospective cohort study of 816 consecutive cases of esophageal ESD performed between 2002 and 2021 at the University of Tokyo Hospital. After 2013, all patients with a diagnosis of superficial esophageal carcinoma covering over 1/2 the esophageal circumference underwent preventive treatment immediately after ESD with either “PGA shielding”, “steroid injection”, or “steroid injection + PGA shielding”. Additional steroid injection was performed for high-risk patients after 2019.

**Results:**

The risk of refractory stricture was especially high in the cervical esophagus (OR 24.77, *p* = 0.002) and after total circumferential resection (OR 894.04, *p* < 0.001). “Steroid injection + PGA shielding” was the only method significantly effective in preventing stricture occurrence (OR 0.36; 95% CI 0.15–0.83, *p* = 0.012). This method also decreased the risk of refractory stricture (OR 0.38; 95% CI 0.10–1.28, *p* = 0.096), but additional steroid injection was the only significantly effective method for prevention of refractory stricture (OR 0.42; 95% CI 0.14–0.98, *p* = 0.029).

**Conclusion:**

Combining steroid injection and PGA shielding is effective for preventing post-ESD stricture and refractory stricture. Additional steroid injection is a viable option for patients at high-risk for refractory stricture.

**Graphical abstract:**

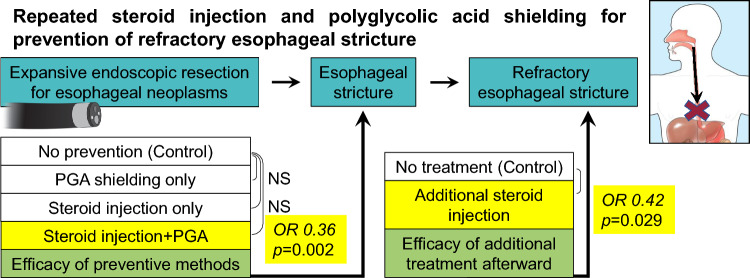

**Supplementary Information:**

The online version contains supplementary material available at 10.1007/s00464-023-10111-z.

Endoscopic submucosal dissection (ESD) is widely performed for the treatment of superficial esophageal carcinoma [1, 2]. However, postoperative stricture frequently occurs after endoscopic resection of over 3/4 the circumference of the esophagus [1, 3], and more recently resection of the cervical esophagus [4–6] and total circumferential resection [6–10] have also been reported to be significant risk factors for postoperative stricture. While oral prednisolone [9, 11] and triamcinolone injection [10, 12, 13] were originally introduced as methods effective in preventing stricture for these high-risk cases, more recently limits in the safety and efficacy of these methods have become apparent [14–17].

Even more problematically, postoperative stricture can become refractory. Refractory strictures do not respond swiftly to standard treatment by endoscopic balloon dilation and may require months or years of treatment [7, 18]. Recent reports have suggested that refractory strictures may be managed by radial incision cutting and/or repeated endoscopic balloon dilation, but these procedures are time-consuming, associated with risks of adverse events such as perforation and hemorrhage [18, 19], and are not always effective. In addition, initiation of steroid treatment after the development of refractory strictures is not effective [20], and therefore identifying cases at high-risk for refractory strictures and initiating prophylactic treatment before development of refractory stricture would be an ideal strategy. However, risk factors and effective methods for the prevention of refractory stricture have yet to be clarified. With an increasing number of patients undergoing endoscopic resection for superficial esophageal carcinoma, this is becoming an increasingly important topic.

Recently, there have been several reports which suggest that combining steroid injection with PGA shielding is effective for the prevention and alleviation of postoperative stricture [6, 21, 22]. In addition, while single-use steroid injection has been reported to be effective for the prevention of stricture [10, 12, 13], and repeated steroid injection has been reported to augment the effects of endoscopic dilation [23], the prophylactic effect of repeated steroid injection into the defect after esophageal ESD has not yet been documented. The first objective of this study was to demonstrate the efficacy of steroid injection, PGA shielding, and the combination of these methods for the prevention of postoperative and refractory stricture after esophageal ESD. The second objective was to demonstrate the efficacy of additional steroid injection thereafter.

## Materials and methods

This is a single-center retrospective cohort study of all cases of esophageal ESD performed at the University of Tokyo between January 1, 2002 and December 31, 2021.

The study complied with the Declaration of Helsinki, and was begun after approval by the Research Ethics Committee of the Graduate School of Medicine and Faculty of Medicine, The University of Tokyo.

### Patients

**Fig. 1:**
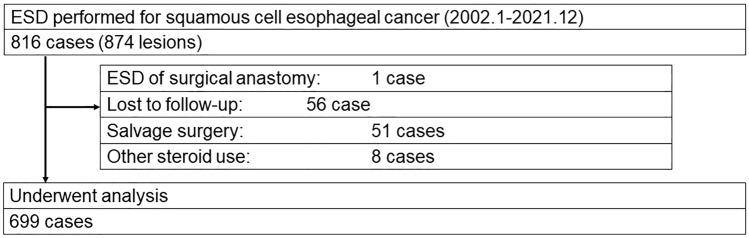
Flowchart of cases included in analysis

From the electronic medical records at the University of Tokyo Hospital, all cases of esophageal ESD performed between January 1, 2002 and December 31, 2021 were identified. A total of 874 neoplasms in 816 consecutive cases of esophageal ESD were extracted. After exclusion of (1) ESD for lesions extending to surgical anastomosis after esophagectomy, (2) cases lost to follow-up within 12 weeks after ESD, (3) cases who underwent salvage surgery after non-curative ESD, (4) cases who used systemic steroids for other reasons, a total of 699 cases were included in analysis (Fig. 1).

### ESD procedure

ESD was performed according to standard ESD procedure [24]. Under either procedural sedation or general anesthesia, close observation of the targeted esophageal lesion(s) was performed. The margin of the lesion was marked using a cutting device of the endoscopists’ choice: either the Dual Knife (KD-630L; Olympus Medical Systems, Co., Tokyo, Japan), ITknife nano (KD-512L/U; Olympus Medical Systems, Co., Tokyo, Japan) or Splash M Knife (DN-D2718B; Pentax Medical, Tokyo, Japan.). A single channel upper gastrointestinal endoscope (GIF Q260J; Olympus Co.) with a high frequency generator VIO 300D (ERBE Elektromedizin GmbH, Tübingen, Germany.) was used, and after submucosal injection of a two-fold diluted solution of 0.4% hyaluronic acid (Mucoup; Johnson and Johnson K.K., Tokyo, Japan.), incision and dissection of the lesion was continued using the cutting device until completion of ESD.

### Prevention of post-ESD stricture

All patients with a preoperative diagnosis of single or multiple adjacent lesions of superficial esophageal cancer covering over 1/2 the circumference of the esophagus and would thereby cause a single post-ESD mucosal defect of over 3/4 of the circumference of the esophagus were defined as “patients at risk of stricture”. Due to lack of public guidelines for prevention of post-ESD stricture, various methods of prevention have been implemented at the University of Tokyo over time (Table 1). Patients who underwent ESD prior to July 2013 did not undergo treatment for prevention of post-ESD stricture. After approval by the Research Ethics Committee of the Graduate School of Medicine and Faculty of Medicine, The University of Tokyo, and trial registration, a single-arm study on the efficacy of PGA shielding has been performed for patients at risk of stricture after July 2013 [25], and combination treatment of steroid injection and PGA shielding has been performed after July 2014 [21]. After completion of these studies, all patients at risk of stricture have undergone combination treatment of steroid injection and PGA shielding until 2018, excluding 2 patients who underwent PGA shielding only because they could not receive steroid injection due to insufficient remaining submucosa. As steroid injection has been established as an effective method of prevention [10, 12, 13], and the added effect of PGA shielding to steroid injection has not been clarified in previous reports [6], steroid injection alone has also become an option at our institute after 2019. While steroid injection is technically easier and comes with less financial burden than PGA shielding, in cases with insufficient remaining submucosa after ESD, steroid injection alone into the muscularis propria has been reported to be a risk factor for subsequent delayed perforation and thus could not be performed in all cases [16, 17]. Due to a trade-off between efficacy and safety, the choice of steroid injection alone was performed at the discretion of the operator according to the condition of the post-ESD defect. As an attempt to further decrease the incidence of stricture, additional steroid injection after ESD has also been introduced after 2019. According to the condition of the post-ESD defect, additional steroid injection was also selected for high-risk cases at the discretion of the operator.

**Table 1:** Prevention of post-ESD stricture at the University of Tokyo

Time period	2002–2013	2013–2014	2014–2019	2019–present
Clinical study	No	UMIN11058	UMIN14642	No
	(Standard therapy)	(Prospective study)	(Prospective study)	(Standard therapy)
Preventive methods immediately after resection				
Low-risk	No	No	No	No
High-risk	No	PGA shielding	Steroid injection + PGA shielding	Steroid injection + PGA shielding
				Steroid injection only
Preventive methods continued after discharge				
Low-risk	No	No	No	No
High-risk	No	No	No	No
				Additional steroid injection

### Immediate steroid injection

In patients who underwent immediate steroid injection, immediately after completion of ESD, a solution of triamcinolone acetonide diluted to a concentration of 5 mg/ml was injected into the submucosal layer of the ESD defect as previously reported [6, 21]. Injection was performed in doses of 0.2 ml per location, for a total of 40–80 mg. For patients undergoing combination therapy, PGA shielding was performed thereafter.

### PGA shielding

In patients who underwent PGA shielding, PGA sheets (Neoveil® NV-M-015G (100 × 50 × 0.15 mm); Gunze Co, Tokyo, Japan) were deployed using either the clip-and-pull method [21, 25, 26] or by delivering multiple patches (approximately 0.5 × 0.5 cm) of PGA through the scope with forceps [22, 27] according to the preference of the operator. After the PGA sheets were delivered to cover the entire circumference of the esophagus, fibrin glue (Beriplast®P Combi-Set; CSL Behring Pharma, Tokyo, Japan.) was then instilled along the entire length of the sheet, firmly fixing the sheets to the post-ESD mucosal defect.

### Perioperative management

On the day before ESD, oral diet was discontinued after the evening meal, and patients were prohibited from eating thereafter until instructed. Oral administration of a daily dose of proton-pump inhibitors was also begun on the day before ESD and continued daily for a minimum of 29 days. The day after ESD, laboratory examinations, along with chest and abdominal X-ray were performed. Oral diet was resumed 2 days after ESD. The patients were followed up for a minimum of 12 weeks to ensure that postoperative stricture would not be overlooked, based on previous reports that postoperative stricture occurs approximately 4 weeks after ESD [25].

### Additional steroid injection

Previous literature has demonstrated that postoperative stricture occurs after a period of 3–4 weeks following ESD in most cases [6]. Thus, in patients who underwent additional steroid injection, a scheduled endoscopic evaluation was performed within 3 weeks after ESD, and a solution of 40 mg triamcinolone acetonide diluted to a concentration of 5–10 mg/ml was injected into the ESD defect. This procedure was repeated at 1–2 week intervals thereafter, until regeneration of the epithelium was confirmed or until the patient could not continue follow-up. Strict follow-up procedures were not implemented due to lack of evidence supporting this method, and intervals were adjusted according to each patient’s physical and social circumstances.

### Endoscopic balloon dilation

Scheduled endoscopic evaluation was not routinely performed within 12 weeks of ESD, except for cases undergoing additional steroid injection. Endoscopic evaluation was usually only performed within 12 weeks of ESD when the patient had a dysphagia score [28] of over 2, which was defined as the condition where the patient could only eat semi-solid foods. Postoperative stricture was defined as the condition where a 9–10 mm diameter upper gastrointestinal endoscope could not pass through the esophageal lumen, and endoscopic balloon dilation (EBD) was only performed if this criteria was met. EBD was initially performed 1 time per week as required until the patient did not meet the criteria for postoperative stricture and could maintain this condition for 4 weeks without intervention. In cases of postoperative stricture found during scheduled additional steroid injection, EBD was performed regardless of symptoms of dysphagia, so that the endoscope could pass through the lumen and additional steroid injection could be performed. An esophageal balloon dilatation catheter (CRE Fixed Wire 12 mm/15 mm/18 mm, Boston Scientific Co, Boston, USA) was used for all EBD procedures.

### Endpoints for analysis

Background factors, endoscopic findings, and histopathological results were extracted from the medical records at the University of Tokyo Hospital. Terminology concerning location and depth were defined in accordance with the Japanese Guidelines for Diagnosis and Treatment of Carcinoma of the Esophagus [1], with cervical esophageal cancer defined as lesions extending to within 18 cm from the incisor teeth. As mentioned above, postoperative stricture was defined as the condition where a 9–10 mm diameter upper gastrointestinal endoscope could not pass through the esophageal lumen. Refractory stricture has previously been defined as the condition where stricture could not be remediated by over 5 sessions at 2-week intervals for a total of 10 weeks [29], with slightly adapted definitions per institute [30]. As EBD was performed at 1-week intervals at our institute, in this study refractory stricture was defined as the condition where over 10 sessions of EBD treatment were required after esophageal ESD. Successful preventive treatment was defined as the condition where postoperative stricture and refractory stricture did not occur within 12 weeks of treatment. To evaluate the efficacy of each method of preventive treatment, the rates of postoperative stricture, and refractory stricture were evaluated.

### Statistical analysis

Risk factors for stricture and refractory stricture were analyzed. Adjusting for possible risk factors for stricture that had been screened by univariable analysis, the odds ratios (OR) for steroid injection and/or PGA shielding, and additional steroid injection were estimated by multivariable logistic regression. Variables that attained a cutoff of 0.05 for univariable *p* value were considered as adjustment variables. Because of (quasi-)complete separation of events among screened variables, some variables’ categories were collapsed, or variables were deleted from regressors if high correlation was observed (e.g., lesion and resection circumferences); further, Firth’s penalized likelihood was utilized to obtain the bias-corrected maximum likelihood estimates from sparse data. All *p*-values and confidence intervals were based on the penalized profile likelihood-ratio test.

The efficacy of each method of prevention was also evaluated by univariable analysis of patients at risk of stricture as described above. All statistical analyses were performed using JMP® Version 16.0 (SAS Institute Inc., Cary, NC, USA).

## Results

### Background factors of patients

**Fig. 2:**
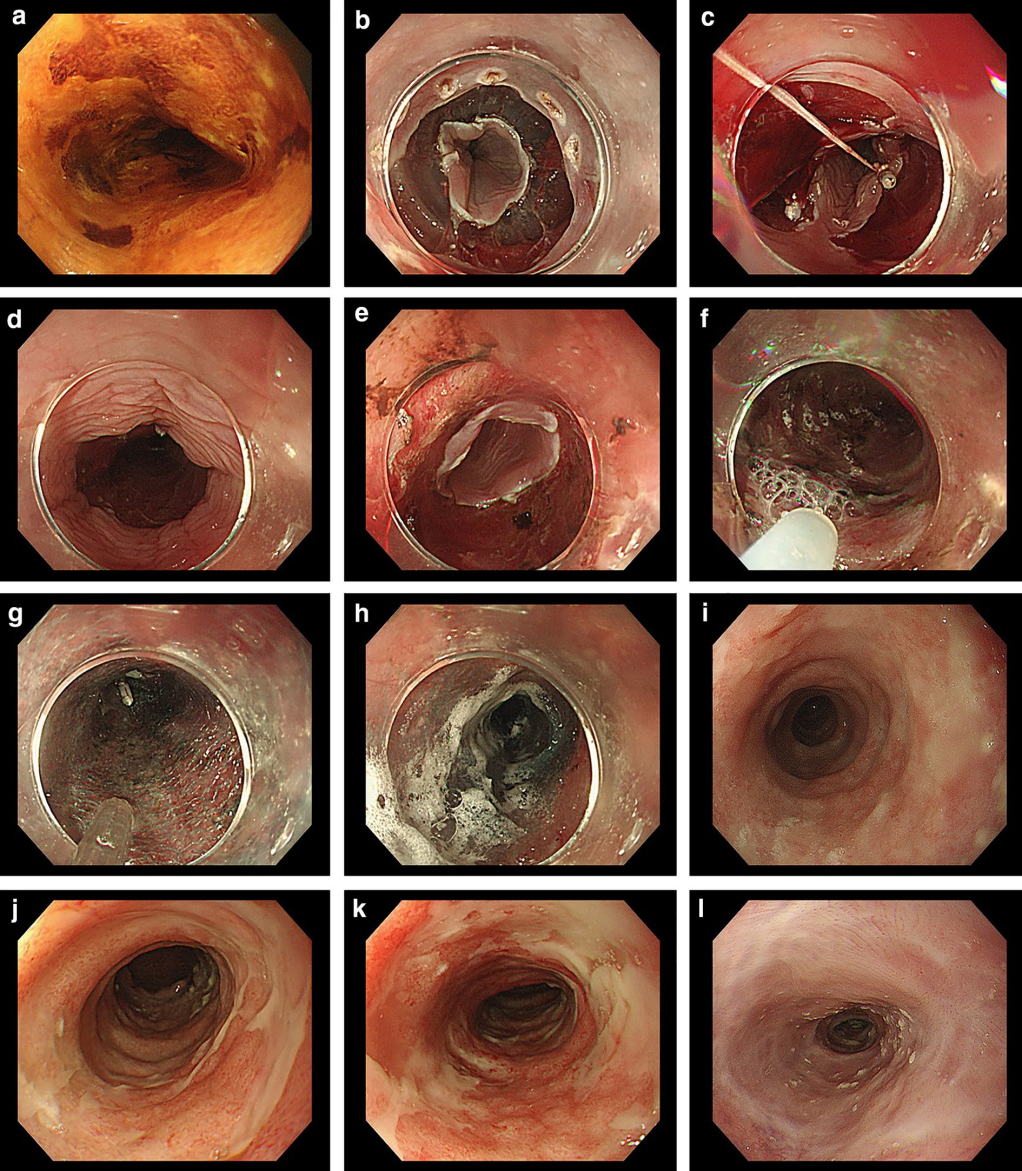
A representative case of steroid injection and PGA shielding and additional steroid injection after total circumferential resection. **a** Endoscopic evaluation with Lugol staining revealed superficial squamous cell carcinoma extending to the entire circumference of the esophagus. **b** After marking the lesion, circumferential incision of the anal side of the lesion was performed. **c** This was followed by incision and submucosal dissection from the oral side. A double-tunnel strategy was employed in this case. **d** Remaining submucosa on the oral side of the defect immediately after ESD was visualized, as well as. **e** Remaining submucosa on the anal side. **f** Triamcinolone was injected evenly into the surface of the submucosa of the defect at approximately 5–10 mm intervals. Special care was taken not to inject into the muscularis propria. **g** A PGA sheet was wrapped around the endoscope and delivered, and after clipping the sheet to the anal side of the defect, the sheet was deployed by simply pulling out the endoscope. **h** The sheet was adhered to the defect with fibrin glue, which bonds within 1 min. **i** The ESD defect 2 weeks after ESD was covered with regenerative tissue and white mucous. Triamcinolone was again injected evenly into the defect at approximately 5–10 mm intervals. **j** This procedure was initially repeated weekly, with signs of epithelialization of the wound beginning at 3 weeks after ESD. **k** Further epithelialization 4 weeks after ESD. **l** Triamcinolone injection was repeated a total of 5 times, until total epithelization was seen 2 months after ESD. Postoperative stricture was successfully prevented in this case

Of the 699 cases included in analysis (Table 2), 86.8% were male, aged 68.7 ± 9.0 years. The majority had a histology of squamous cell carcinoma (95.7%), with the remaining comprised adenocarcinoma and basaloid carcinoma. While adenocarcinoma and basaloid carcinoma were mostly located in the lower thoracic esophagus or abdominal esophagus, there was no difference in other background characteristics between the different histology. A representative case is shown in Fig. 2.

**Table 2:** Background factors of cases with superficial esophageal carcinoma

	ALL	Squamous cell carcinoma	Other
			(adenocarcinoma, basaloid)
Patient factors	*n* = 699	*n* = 669	*n* = 30
Age years, mean ± SD	68.7 ± 9.0	68.9 ± 9.0	65.5 ± 9.6
Gender Male/Female (Male%)	607/92 (86.8)	577/92 (86.2)	30/0 (100)
Lesion factors*			
Location			
Location Ce *n* (%)	30 (4.3)	30 (4.5)	0 (0)
Location Ut *n* (%)	83 (11.8)	82 (12.3)	1 (3.3)
Location Mt *n* (%)	407 (58.2)	407 (60.8)	0 (0)
Location Lt *n* (%)	145 (20.7)	139 (20.8)	6 (20.0)
Location Ae *n* (%)	34 (4.9)	11 (1.6)	23 (76.7)
Depth			
M *n* (%)	M 650 (94.1)	EP 231 (34.9)	EP 4 (13.3)
		LPM 322 (48.7)	SMM (11 (36.7)
		MM 70 (10.6)	LPM 3 (10.0)
			DMM 9 (30.0)
SM *n* (%)	SM 41 (5.9)	SM1 9 (1.4)	SM1 1 (3.3)
		SM2 28 (4.2)	SM2 2 (6.7)
		SM3 1 (0.2)	SM3 0 (0.0)
Previous treatment to same location (EMR,ESD,RT)**	68 (9.7)	65 (9.7)	3 (11.1)
Lesion size mm, mean ± SD	21.3 ± 13.4	21.4 ± 13.2	19.2 ± 17.7
Lesion circumference	*Data missing for 1	*Data missing for 1	
≦50% n (%)	545 (78.1)	520 (77.8)	25 (83.3)
51–75% n (%)	110 (15.8)	107 (16.0)	3 (10.0)
76–99% n (%)	29 (4.2)	29 (4.3)	0 (0.0)
100% n (%)	14 (2.0)	12 (1.8)	2 (6.7)
Treatment factors			
Resection size mm, mean ± SD	35.3 ± 13.7	34.9 ± 12.9	44.3 ± 24.3
Resection circumference	*data missing for 1	*data missing for 1	
≦50% n (%)	378 (54.2)	362 (54.2)	16 (53.3)
51–75% n (%)	168 (24.1)	161 (24.1)	7 (23.3)
76–99% n (%)	135 (19.3)	130 (19.5)	5 (16.7)
100% n (%)	17 (2.4)	15 (2.2)	2 (6.7)
Operation time min, mean ± SD	76.7 ± 48.6	75.4 ± 46.9	104.1 ± 72.5
Prophylactic treatment immediately after ESD***			
No *n* (%)	505 (72.2)	482 (72.0)	23 (76.7)
PGA *n* (%)	53 (7.6)	51 (7.6)	2 (6.7)
Steroid injection n (%)	36 (5.2)	33 (4.9)	3 (10.0)
Steroid injection + PGA n (%)	105 (15.0)	103 (15.4)	2 (6.7)
Additional steroid injection n (%)	48 (6.9)	45 (6.7)	3 (10.0)

*Lesion factors were defined in accordance with the Japanese Guidelines for Diagnosis and Treatment of Carcinoma of the Esophagus

*Ce* cervical, *Ut* upper thoracic, *Mt* middle thoracic, *Lt* lower thoracic, *Ae* abdominal, *M* invasion depth limited to the mucosal layer, *SM* invasion into the submucosa, *EP* epithelial, *LPM* lamina propria, *MM* muscularis mucosa, *SMM* superficial muscularis mucosa, *DMM* deep muscularis mucosa

**EMR endoscopic mucosal resection, *ESD* endoscopic submucosal dissection, *RT* radiotherapy

***PGA polyglycolic acid shielding

### Risk factors associated with postoperative stricture and refractory stricture

Of the factors associated with the incidence of postoperative stricture in univariable analysis (Supplementary Table 1), multivariable logistic regression analysis (Table 3) demonstrated that the location of the lesion (*p* = 0.089) and the resection circumference (*p* < 0.001) were the only risk factors associated with postoperative stricture. Concerning location, the risk of postoperative stricture was especially high in the cervical esophagus (OR 2.05, *p* = 0.303) and upper thoracic esophagus (OR 2.82, *p* = 0.037). Concerning resection circumference, the risk of postoperative stricture increased in proportion to resection circumference, with significant risk after resection of over 1/2 the circumference (OR 12.56, *p* < 0.001), and extremely high-risk after total circumferential resection (OR 605.66, *p* < 0.001).

**Table 3:** Multivariable logistic regression model on risk factors associated with stricture occurrence

	Adjusted OR (95% CI)	*p* value
Lesion factors*		
Location		0.089
Location Ce	2.05 (0.53–7.54)	0.303
Location Ut	2.82 (1.05–7.62)	0.037
Location Mt	1.14 (0.56–2.40)	0.726
Location Lt	1.00 (Reference)	
Location Ae	0.41 (0.06–1.94)	0.230
Depth		0.358
M	1.00 (Reference)	
SM	1.31 (0.76–2.15)	
Treatment factors		
Resection circumference		< 0.001
≦50%	1.00 (Reference)	
51–75%	12.56 (3.79–63.8)	< 0.001
76–99%	78.91 (23.00–411.88)	< 0.001
100%	605.66 (101.10–5184.78)	< 0.001
Operation time		0.515
≦30 min	1.00 (Reference)	
31–60 min	3.12 (0.30–423.65)	0.269
61–90 min	5.15 (0.50–699.17)	0.121
91–120 min	3.87 (0.37–525.79)	0.197
≧121 min	4.71 (0.45–641.37)	0.143
Prophylactic treatment immediately after ESD**		0.062
No	1.00 (Reference)	
PGA	0.94 (0.37–2.30)	1.000
Steroid injection	0.83 (0.26–2.36)	0.712
Steroid injection + PGA	0.36 (0.15–0.83)	0.012
Additional steroid injection	1.53 (0.99–2.36)	0.048

All *p*-values and confidence intervals were based on the penalized profile likelihood-ratio test

*Lesion factors were defined in accordance with the Japanese Guidelines for Diagnosis and Treatment of Carcinoma of the Esophagus

*Ce* cervical, *Ut* upper thoracic, *Mt* middle thoracic, *Lt* lower thoracic, *Ae* abdominal

M: invasion depth limited to the mucosal layer, SM: invasion into the submucosa

**PGA polyglycolic acid shielding

Similar tendencies were found for risk factors associated with refractory stricture (Table 4). Concerning location, the risk of refractory stricture was significantly high in the cervical esophagus (OR 24.77, *p* = 0.002). The risk of refractory stricture increased in proportion to resection circumference, with a significant risk of refractory stricture after resection of over 3/4 the circumference (OR 158.31, *p* < 0.001), and extremely high-risk after total circumferential resection (OR 894.04, *p* < 0.001).

**Table 4:** Multivariable logistic regression model on factors associated with refractory stricture

	Adjusted OR (95% CI)	*p* value
Lesion factors*		
Location		0.007
Location Ce	24.77 (2.96–278.83)	0.002
Location Ut	6.01 (1.29–34.54)	0.016
Location Mt	1.82 (0.45–9.11)	0.400
Location Lt	1.00 (Reference)	
Location Ae	4.05 (0.41–40.69)	0.248
Depth		0.921
M	1.00 (Reference)	
SM	0.86 (0.28–2.03)	0.921
Treatment factors		
Resection circumference		< 0.001
≦50%	1.00 (Reference)	
51–75%	2.89 (0.01–1098.52)	1.000
76–99%	158.31 (14.45–41,810.38)	< 0.001
100%	894.04 (267,884.18)	< 0.001
Operation time		0.590
≦30 min	1.00 (Reference)	
31–60 min	0.35 (0.01–65.72)	1.000
61–90 min	0.75 (0.02–146.38)	1.000
91–120 min	0.22 (0.00–45.87)	1.000
≧121 min	0.81 (0.02–160.00)	1.000
Prophylactic treatment immediately after ESD**		0.450
No	1.00 (Reference)	
PGA	0.44 (0.08–1.88)	0.229
Steroid injection	0.59 (0.05–4.22)	0.623
Steroid injection + PGA	0.38 (0.10–1.28)	0.096
Additional steroid injection	0.42 (0.14–0.98)	0.029

All *p*-values and confidence intervals were based on the penalized profile likelihood-ratio test

*Lesion factors were defined in accordance with the Japanese Guidelines for Diagnosis and Treatment of Carcinoma of the Esophagus

*Ce* cervical, *Ut* upper thoracic, *Mt* middle thoracic, *Lt* lower thoracic, *Ae* abdominal

*M* invasion depth limited to the mucosal layer, *SM* invasion into the submucosa

**PGA polyglycolic acid shielding

### Efficacy of methods of prevention

There were no intra-procedural and peri-procedural adverse events related to any of the methods of prophylactic treatment.

Concerning the efficacy of methods of prevention, differences were found in the efficacy of prophylactic treatment performed immediately after ESD (*p* = 0.062), with “steroid injection + PGA shielding” being the only method significantly effective in preventing stricture (OR 0.36; 95% CI 0.15–0.83, *p* = 0.012). This method had a higher effect than only “steroid injection” (OR 0.83, *p* = 0.712) or “PGA shielding” (OR 0.94, *p* = 1.000) alone. A higher rate of stricture was seen after additional steroid injection (OR 1.53, *p* = 0.048).

Concerning the risk of refractory stricture, a similar tendency was found, with “steroid injection + PGA shielding” seeming to be the most effective method (OR 0.38; 95% CI 0.10–1.28, *p* = 0.096) among the methods performed immediately after ESD. Contrary to the negative effect found for prevention of stricture, additional steroid injection was significantly effective in preventing refractory stricture (OR 0.42; 95% CI 0.14–0.98, *p* = 0.029). Unadjusted odds ratios and relative risk for patients with resection of over 3/4 the circumference of the esophagus (Supplementary Table 2) have been included for reference.

## Discussion

We have previously demonstrated that the combination of steroid injection and PGA shielding is effective for safely preventing stricture after esophageal ESD, with significant reduction in incidence and severity of postoperative stricture [6, 21, 25]. For the first time, we have demonstrated that there is a synergic effect in combining these 2 methods, decreasing the incidence of postoperative stricture more significantly than either of these methods alone. In addition, the combination of these methods may also decrease the incidence of refractory stricture, which is an even more important clinical issue.

Interestingly, neither of these methods alone displayed a significant effect for either the prevention of postoperative stricture occurrence or refractory stricture. However, steroid injection has already been established as an effective method of stricture prevention [10, 12, 13], and these results may have been influenced by the small sized cohort. These results seem to only signify that a larger number-needed-to-treat is required for each of these methods alone.

More interestingly, the combination of steroid injection and PGA was effective in preventing refractory stricture, which suggests that the clinical effect of this method lasted for at least several weeks. This is consistent with previous reports on the long-lasting effect of PGA [31]. Meanwhile, single-use steroid injection performed only immediately after ESD seems to have a more limited effect on refractory stricture, which may signify that the effects of steroid injection are short-lasting.

Another key point of interest is that in patients undergoing additional steroid injection, the ratio of patients requiring EBD, and thus meeting the definition of postoperative stricture, increased contrary to expectation. However, in all the patients in this group, EBD became necessary before symptoms of dysphagia developed. This was because it was necessary to perform EBD in cases with a narrow lumen, so that the endoscope could pass and additional steroid injection could be performed. This implies that for patients undergoing additional steroid injection, pre-clinical stricture may have been detected during scheduled endoscopy, while similar pre-clinical stricture was not detected in other patients because they did not undergo scheduled endoscopy. Thus these results do not necessarily mean that additional steroid injection increases the risk of stricture. Contrarily, additional steroid injection was the only method which significantly decreased the risk of refractory stricture, which is as previously mentioned, a much more important clinical issue. This may be explained by the long-lasting effect of repeated therapy. While this method requires multiple sessions of endoscopic therapy and requires EBD in pre-clinical stricture cases with a narrow lumen and thus is associated with substantial patient burden, additional steroid injection is a very viable option for patients at high-risk for refractory stricture.

However, due to differences in background factors of the patients included in various studies [6], risk factors for refractory stricture have not been fully clarified. Through the multivariable logistic regression analysis performed in this study, the most significant risk factors for postoperative stricture occurrence and refractory stricture; location and resection circumference, have been reconfirmed and are consistent with previous reports [4, 5, 8, 9]. The combination of steroid injection and PGA shielding, and additional steroid injection should be especially considered for patients with lesions located in the cervical esophagus and total circumferential lesions. However, although this method is to date one of the few methods with the potential to successfully prevent postoperative stricture after total circumferential ESD [32, 33], this method is not effective enough to consistently prevent postoperative stricture in all high-risk cases. In addition, while the prophylactic methods employed in this study were safe with no adverse events, they require technical expertise and repeated steroid injection may not be always possible depending on the patients’ social circumstances. There is a need for the development of even more effective methods for the prevention of postoperative stricture and refractory stricture.

There were several limitations to this study. First, this was a single-center retrospective cohort analysis involving only a limited number of patients. Due to the retrospective study design, this study is subject to multiple biases. To minimize the effect of these biases, the authors have conducted a multivariable logistic regression analysis, yet there is a possibility that significant biases remain. If possible a multi-center randomized controlled study is desirable. Second, although the rate of stricture occurrence in cervical esophageal cancer patients was markedly higher than in other locations, the number of patients in this study was not sufficient to demonstrate a statistically significance. However when dilation of the cervical esophagus is required to treat stricture there is a risk of airway obstruction [34], and thus prevention of stricture in the cervical esophageal cancer has a high priority from a clinical standpoint. Third, because the remaining mucosa contracts after resection, evaluation of resection circumference is subjective. However, current guidelines [1, 3] are based on literature which have assessed the risk of postoperative stricture based on the resection circumference [3, 35], and thus the same criteria was employed in this study [7]. Finally, several of the patients with refractory stricture in this study still require EBD, and the number of EBD sessions in this study may be underestimated. Further follow-up is required.

## Conclusion

The combination of triamcinolone injection and the shielding method with PGA sheets and fibrin glue is more effective than either of these methods alone for preventing and alleviating stricture after esophageal ESD. Additional triamcinolone injection is effective for prevention of refractory stricture and may be a viable option for high-risk cases.

## Supplementary Information

Below is the link to the electronic supplementary material.

Supplementary file1 (DOCX 34 KB)

Supplementary file2 (DOCX 32 KB)

## Data Availability

The data, analytic methods, and study materials will be made available to other researchers upon reasonable request to the corresponding author.
