# Supplementary material for: Repeated steroid injection and polyglycolic acid shielding for prevention of refractory esophageal stricture
Source: Surg Endosc. 2023 May 16;37(8):6267–77. doi: 10.1007/s00464-023-10111-z (PMC10338585; doi:10.1007/s00464-023-10111-z)
Supplement: Supplementary file 1 — Supplementary file1 (DOCX 34 KB) [file 464_2023_10111_MOESM1_ESM.docx]

Supplementary Table 1: Univariable analysis on risk factors associated with stricture occurrence

|  |  | No stricture | Stricture | *p* value |
| --- | --- | --- | --- | --- |
| **Patient factors** | | n=614 | n=85 |  |
| Age years, mean±SD | | 68.7±9.1 | 68.8±8.4 | 0.815 |
| Gender Male/Female (Male%) | | 531/83 (86.5) | 76/9 (89.4) | 0.607 |
| **Lesion factors *** | | | | |
| Location | |  |  | 0.040 |
|  | Location Ce n (%) | 23/30 (76.7) | 7/30 (23.3) |  |
|  | Location Ut n (%) | 66/83 (79.5) | 17/83 (20.5) |  |
|  | Location Mt n (%) | 367/407 (90.2) | 40/407 (9.8) |  |
|  | Location Lt n (%) | 127/145 (87.6) | 18/145 (12.4) |  |
|  | Location Ae n (%) | 31/34 (91.2) | 3/34 (8.8) |  |
| Depth* | |  |  | 0.328 |
|  | M n (%) | 572/650 (88.0) | 78/650 (12.0) |  |
|  | SM n (%) | 34/41 (82.9) | 7/41 (17.1) |  |
| Previous treatment to same location (EMR,ESD,RT) ** | | 59/614 (9.6) | 9/85 (10.6) | 0.778 |
| Lesion size mm, mean±SD | | 19.4±12.2 | 34.7±14.2 | <0.001 |
| Lesion circumference | |  |  | <0.001 |
|  | ≦50% n (%) | 522/545 (95.8) | 23/545 (4.2) |  |
|  | 51-75% n (%) | 75/110 (68.2) | 35/110 (31.8) |  |
|  | 76-99% n (%) | 14/29 (48.3) | 15/29 (51.7) |  |
|  | 100% n (%) | 2/14 (14.3) | 12/14 (85.7) |  |
| **Treatment factors** | |  |  |  |
| Resection size mm, mean±SD | | 33.8±13.0 | 46.0±13.4 | <0.001 |
| Resection circumference | | | | <0.001 |
|  | ≦50% n (%) | 376/378 (99.5) | 2/378 (0.5) |  |
|  | 51-75% n (%) | 152/168 (90.5) | 16/168 (9.5) |  |
|  | 76-99% n (%) | 82/135 (60.7) | 53/135 (39.3) |  |
|  | 100% n (%) | 3/17 (17.7) | 14/17 (82.4) |  |
| Operation time min, mean±SD | | 70.5±42.7 | 121.6±63.9 | <0.001 |
| Prophylactic treatment immediately after ESD*** | | | | <0.001 |
|  | No n (%) | 473/505 (93.7) | 32/505 (6.4) |  |
|  | PGA n (%) | 38/53 (71.7) | 15/53 (28.3) |  |
|  | Steroid injection n (%) | 30/36 (83.3) | 6/30 (16.7) |  |
|  | Steroid injection + PGA n (%) | 73/105 (69.5) | 32 (30.5) |  |
| Additional steroid injection n (%) | | 30/48 (62.5) | 18/48 (37.5) | <0.001 |

* Lesion factors were defined in accordance with the Japanese Guidelines for Diagnosis and Treatment of Carcinoma of the Esophagus

M: invasion depth limited to the mucosal layer, SM: invasion into the submucosa.

** EMR: endoscopic mucosal resection, ESD: endoscopic submucosal dissection, RT: radiotherapy

***PGA: polyglycolic acid shielding
