# Supplementary material for: Repeated steroid injection and polyglycolic acid shielding for prevention of refractory esophageal stricture
Source: Surg Endosc. 2023 May 16;37(8):6267–77. doi: 10.1007/s00464-023-10111-z (PMC10338585; doi:10.1007/s00464-023-10111-z)
Supplement: Supplementary file 2 — Supplementary file2 (DOCX 32 KB) [file 464_2023_10111_MOESM2_ESM.docx]

Supplementary Table 2: Unadjusted risk assessment for stricture occurrence and refractory stricture after resection of over 3/4 the circumference

| **Risk of stricture occurrence for resection over 3/4 of the circumference** | | | | |
| --- | --- | --- | --- | --- |
|  |  | OR (95% CI) | RR (95% CI) | *p* value |
| Prophylactic treatment (no/PGA/steroid+PGA) ** | | |  |  |
|  | no | 1.00 (reference) | 1.00 (reference) |  |
|  | PGA | 1.06 (0.39-2.88) | 1.02 (0.66-1.61) | 1.000 |
|  | steroid injection | 0.68 (0.16-2.94) | 0.82 (0.37-1.81) | 0.718 |
|  | steroid injection + PGA | 0.47 (0.21-1.03) | 0.66 (0.43-0.99) | 0.070 |
| Additional steroid injection | | 0.66 (0.30-1.47) | 0.79 (0.48-1.29) | 0.330 |
| **Risk of refractory stricture for resection over 3/4 of the circumference** | | | | |
|  |  | OR (95% CI) | RR (95% CI) | *p* value |
| Prophylactic treatment (no/PGA/steroid+PGA) | | |  |  |
|  | no | 1.00 (reference) | 1.00 (reference) |  |
|  | PGA | 0.34 (0.08-1.37) | 0.41 (0.12-1.35) | 0.207 |
|  | steroid injection | 0.34 (0.04-3.05) | NA* | 0.327 |
|  | steroid injection + PGA | 0.44 (0.17-1.15) | 0.52 (0.24-1.10) | 0.120 |
| Additional steroid injection | | 0.27 (0.06-1.21) | 0.31 (0.08-1.26) | 0.108 |
| **Number of balloon dilation sessions for resection over 3/4 of the circumference** | | | | |
|  |  |  | EBD sessions | *p* value |
| Prophylactic treatment (no/PGA/steroid+PGA) ** | | |  | 0.186 |
|  | no |  | 5.54±7.75 |  |
|  | PGA |  | 4.15±6.98 |  |
|  | steroid injection |  | 2.44±3.88 |  |
|  | steroid injection + PGA |  | 3.53±7.31 |  |
| Additional steroid injection | |  |  | 0.098 |
|  | no |  | 4.57±7.34 |  |
|  | yes |  | 2.24±6.47 |  |

* not calculable due to null event

**PGA: polyglycolic acid shielding
